# SVAS^3^: Strain Vector Aided Sensorization of Soft Structures

**DOI:** 10.3390/s140712748

**Published:** 2014-07-17

**Authors:** Utku Culha, Surya G. Nurzaman, Frank Clemens, Fumiya Iida

**Affiliations:** 1 Bio-Inspired Robotics Lab, Department of Mechanical and Process Engineering, ETH Zürich, 8092 Zurich, Switzerland; E-Mails: surya.nurzaman@mavt.ethz.ch (S.G.N.); fumiya.iida@mavt.ethz.ch (F.I.); 2 EMPA Dübendorf, Überlandstrasse 129, 8600 Dübendorf, Switzerland; E-Mail: Frank.Clemens@empa.ch

**Keywords:** sensor morphology design, soft strain gauges, conductive thermoplastic elastomer, soft robotics, wearable electronics

## Abstract

Soft material structures exhibit high deformability and conformability which can be useful for many engineering applications such as robots adapting to unstructured and dynamic environments. However, the fact that they have almost infinite degrees of freedom challenges conventional sensory systems and sensorization approaches due to the difficulties in adapting to soft structure deformations. In this paper, we address this challenge by proposing a novel method which designs flexible sensor morphologies to sense soft material deformations by using a functional material called conductive thermoplastic elastomer (CTPE). This model-based design method, called Strain Vector Aided Sensorization of Soft Structures (SVAS^3^), provides a simulation platform which analyzes soft body deformations and automatically finds suitable locations for CTPE-based strain gauge sensors to gather strain information which best characterizes the deformation. Our chosen sensor material CTPE exhibits a set of unique behaviors in terms of strain length electrical conductivity, elasticity, and shape adaptability, allowing us to flexibly design sensor morphology that can best capture strain distributions in a given soft structure. We evaluate the performance of our approach by both simulated and real-world experiments and discuss the potential and limitations.

## 1. Introduction

Soft materials are capable of high deformations and conformity to unstructured forms which makes them interesting and useful for robotic applications [1,2]. These soft bodied robots can flexibly deform and significantly change their shapes to accomplish tasks like locomotion in unstructured environments or manipulation of complex objects. Some examples of the recent achievements in soft robotics research area include a soft gripper capable of picking up unfamiliar objects with widely varying shape and surface [3], a soft rolling robot inspired by a caterpillar's ability to roll over uneven terrains [4], a robotic arm modeled based on the characteristic muscles of the octopus [5] and a soft robot capable of squeezing itself through obstacles by changing its gait pattern [6].

Although soft materials enable complex and rich behaviors, the fact they have an almost infinite amount of degrees of freedom challenges soft robotics in terms of sensorization of the soft bodies to sense the environment or its own spatial configuration. One of the suggested solutions to evaluate structure curvature was based on external optical sensors [7–11]. However, as it is not always possible to have a structured environment with external optical sensors like cameras, recently, alternative approaches which relied on embedding sensors in the soft structures have been proposed [12]. Mainly driven by tactile sensing [13–15] and bio-medical applications [16], there has been important research on soft sensors. While some of these sensors can detect one type of stimulus like multi-axis strain [17,18], there has been studies which show multimodal sensing such as pressure and force [19,20], shear and normal force [21,22] and strain and pressure [23].

Despite these highly stimulating works, the role of the size, shape and placement of the sensors, commonly known as sensor morphology, does not seem to have been thoroughly investigated. While the importance of sensor morphology in determining the sensing characteristics and performance has gained a lot of attention in biology [24,25], embodied intelligence [26], and most recently in robotics [27,28], its role in soft body sensing still remains as a challenge. The sensorization solutions so far have had the potential for enabling customizable sensor morphology, but required complex molding and suggested casting processes for integration [13,29] or realized commonly used sensor morphologies [30,31].

**Figure 1.**
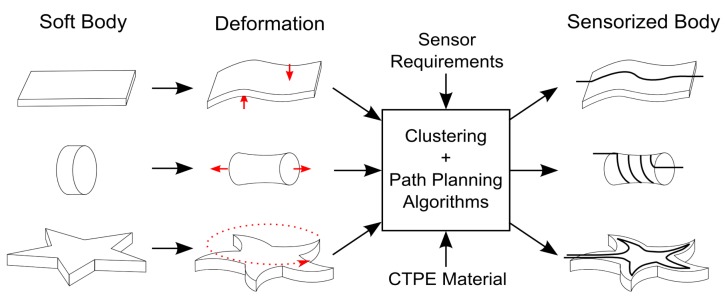
A conceptual schematics of the SVAS3 approach. Three examples of soft bodies are deformed (shown with red arrows) and sensorized with CTPE-based sensors (shown with black curves).

In this paper we propose a technological solution which we call SVAS^3^ to sensorize soft and deformable bodies with flexible and easily integrable sensor morphology, as shown in Figure 1. The proposed technological solution emphasizes two aspects for soft structure sensorization: the exploitation of strain information within soft deformations for the design of characteristic sensor morphologies and the usage of a soft, elastic and easily customizable strain gauge sensor system which can realize these morphologies. The former aspect depends on the generation of strain when a soft structure undergoes deformation. In our method, we can model soft structures and deformations to extract strain information to localize characteristic strain regions on the structure surface. These regions are used as a template to design morphologies for flexible strain gauge sensors. The latter requires a suitable sensor material which can be used to comply with the designs generated by our approach. Out of many possible state-of-the-art sensor materials to fabricate strain sensors like liquid metals [13,17], carbon or metal coated yarns [32,33], carbon nano-tube films [34], in our paper we have decided to use a specific type of a carbon filler-containing polymer composite [35–37] because these structures can reach strain lengths above 100%. The sensors in this approach are fabricated with a Conductive Thermoplastic Material (CTPE), which can be produced quickly and flexibly in terms of shape and size [38]. CTPE has thermoplastic properties that enable the fabrication of different sensor morphologies with simple methods, like heated extrusion or injection molding, which also allows the sensors to be quickly integrated into various soft objects in a modular and therefore intrinsically scalable way. The elastic and electrical properties of the fabricated sensors, e.g., linear response over a wide range of strain lengths, let us easily model them and estimate their performance through the design algorithms in the SVAS^3^ approach. In this paper, in order to show the efficacy and scalability of our suggested approach, we design sensor morphologies to discriminate final postures of soft structures due to bending, twisting and pushing deformations, and evaluate these designs by integrating CTPE-based strain sensors on physical platforms. We also present a sample application on a latex glove to discriminate hand signs in order to show that our method can be used in research fields in wearable electronics and smart textiles in addition to soft robotics.

The remaining structure of this paper is presented as follows: in Section 2, we will introduce the conductive thermoplastic elastomer material and how it can be fabricated to create strain sensors. In Section 3, we will introduce the design approach and present its properties. In Section 4, we will use our suggested method to design sensor morphologies and perform simulation and real world experiments. In Section 5 we will evaluate our approach and discuss a possible application based on soft body (gloves) with integrated fiber sensor structures. Finally, we will conclude the work and list several relevant future works in Section 6.

## 2. Conductive Thermoplastic Elastomer for Strain Sensing

In our approach we use a conductive thermoplastic elastomer (CTPE) developed by EMPA [38] for giant strain sensing, e.g., above 100% reversible strain length. The material is based on a commercial thermoplastic elastomer matrix filled with 50 wt% carbon black powder which makes this hybrid a candidate for a piezoresistant sensor material. This composition is mixed in a high shear mixer to blend the polymer with the inorganic conductive powder at a temperature of 180 °C. The extracted compound has conductive, thermoplastic and elastic properties which are exploited during fabrication of the sensors, as well as in the sensing mechanism itself.

The carbon black ingredient of the material generates a percolated network inside the CTPE which results in electrical conductivity throughout the material body. When strain is applied, the percolation network is changing due to rotation of non-spherical carbon black agglomerates which are still present in the polymeric matrix. This rotation is reversible and the hybrid material can be therefore used for strain sensing in a giant displacement range. CTPE-based sensors are only responsive to strain due to this formulation, but their morphology and placement on target structures can enable the sensing of other stimuli as long as a mapping between the applied stimuli and the strain they generate can be expressed. Additionally, CTPE strain gauges have an almost linear response to applied strain, which makes them a suitable option for easy modeling in our approach.

**Figure 2.**
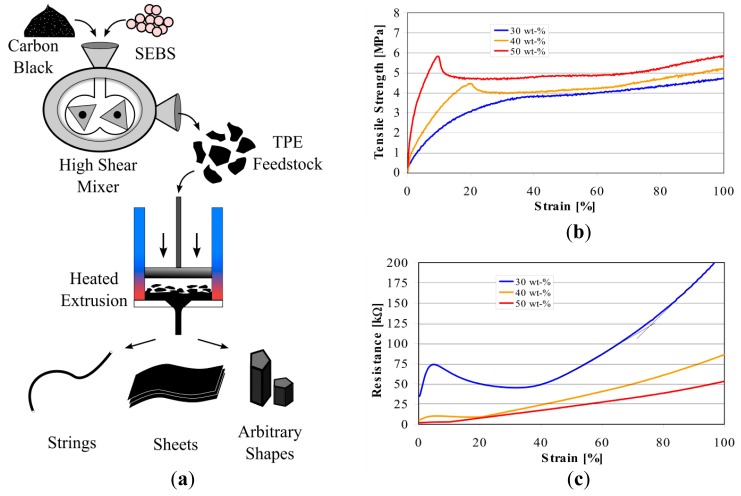
Thermoplastic and mechanical properties of CTPE. (**a**) Fabrication process that can easily generate arbitrary forms; (**b**) Mechanical and (**c**) electrical properties of CTPE when shaped into fibers adapted from previous work by EMPA [38].

Thermoplasticity comes into play when custom shaped sensors need to be fabricated for complex surfaces, while elasticity allows the sensors to undergo high deformations. Figure 2a shows that when the hybrid sensor material is extracted from the high shear mixer, it can be fed to warm presses or heated extruders to fabricate variable sizes and centro-symmetric shapes such as fibers, tubes and sheets. More complex shapes can be created with laser cutters, 3D printers, injection molding or even hand crafting. Such custom shaped elastic sensors can easily conform to deformable continuum body surfaces to acquire more accurate information.

Figure 2b,c shows the mechanical and electrical properties of the CTPE with different carbon content ratios when it is morphed into fiber shapes. It can be seen that sensors become more brittle and stiffer with the addition of carbon black into their structure, which is an expected outcome as the elasticity and softness of the thermoplastic elastomer material is being altered by the stiff unplasticised carbon powder. With low carbon content (30 wt%), the sensor is softer compared to higher carbon content which additionally introduce yield points in the force-strain curve. On the other hand, the carbon content also influences the electrical properties. While the sensor material with low carbon content has complex and separable phases, the response of the sensor with respect to strain becomes smoother and linear with high carbon content. One very unique property of the developed piezoresistant material is the independence between force (or stress) and electrical resistivity. Therefore strain of structures can be directly measured if stiffness of soft body structure is higher in comparison to the piezoresistive sensor. The effect of carbon content on sensor characteristics is explained in previous work by EMPA [38] and Flandin *et al.* [39]. It is worthwhile to mention that direct comparison with carbon filled hybrids in the literature is difficult because percolation behavior, conductivity and maximum strain depend on the carbon and the polymeric matrix material.

For our approach, we have used a CTPE material with 50 wt% carbon content and the heated extrusion method to fabricate strain gauge sensors in fiber shape with 0.3 mm diameter. The resulting fibers had approximately 2 MPa of Young's modulus, with a base resistivity of 37.5 Ω/mm and showed an almost linear response to strain with an average value of 0.66 kΩ/mm ± 13%. In our paper we focus on the design of sensor morphologies with desired sensing characteristics, therefore we preferred to concentrate on the electro-mechanical properties of the chosen sensor technology in simulation models and experiments. Previous work by EMPA covers intensively other technical properties of the material and fabricated sensors such as hysteresis (experiments show a low hysteresis of 2.25% over 80% strain working range), repeatability, sensor drifting and effects of long term usage [38,40].

## 3. SVAS^3^ Design Method

In continuum mechanics, when a force is applied to a solid material it undergoes a deformation, whose mechanical properties can be analyzed with the relationship between the stress in the body that the force causes and the strain that occurs during the deformation of the body [41]. In classical terms, this relation can be expressed with Hooke's Law:

(1) σ=F/A=Eɛ

where the stress σ is generated by the force *F* on a cross section of *A* on the material. The resulting strain ε is dependent on the elastic properties of the material, and can be dictated by its Young's modulus *E*, as long as the material shows elastic and reversible deformation while the applied stress is below the yield stress.

Deformations in soft bodies can also be explained by the same formula as long as the structure does not exhibit plastic deformations. Following this idea, we hypothesize that for every complex deformation, there exists a unique and representative strain information. In our approach, we use this strain information and its geometric properties to design morphologies for flexible sensors which are responsive to strain. Sensor morphologies are designed by following five consecutive steps: (1) soft body and elastic deformation modeling; (2) strain vector extraction; (3) strain region clustering and (4) path planning for final morphology formation. These four steps end up with a final strain path where fiber shaped sensors, which are fabricated with CTPE material, can be placed on to gather strain information and estimate the sensor response to the selected deformations.

### 3.1. Soft Body Modeling

The overall approach starts with the modeling of the soft structure and the deformations that generate the strain information which will be used to construct the final sensor morphology. For modeling, we are using an open source platform called VoxCad [42] that provides a computationally efficient simulation environment for soft structures. In VoxCad, a mesh of discrete 3D pixels, *i.e.*, voxels, which are connected to each other with spring damper systems, are used to construct larger complex soft structures. These voxels can be given configurable material properties such as elasticity, density and thermal expansion, which allow the definition of the statics and dynamics behaviors of the final objects. The dynamics and non-linear complex deformations of these structures can be simulated by the usage of external forces and constraints. Constraints such as self-collisions or anchoring points enable the computation of complex postures due to large deformations and interactions between several objects.

**Figure 3.**
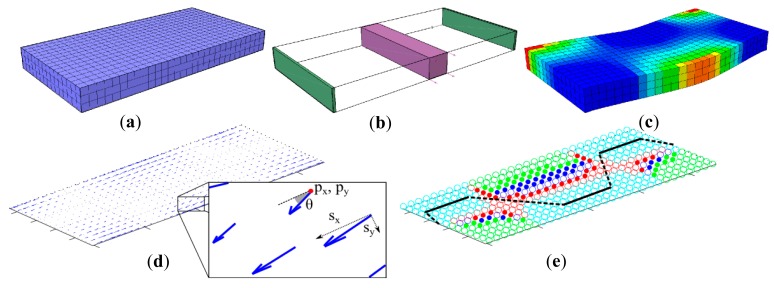
Overall process of SVAS^3^ explained with an example soft structure block. (**a**) Soft body constructed with voxels; (**b**) constraints and stimulus applied and (**c**) soft body deformation simulated; (**d**) Strain vectors are extracted from deformations and (**e**) clustered to generate the final sensor morphology.

In Figure 3a, a prismatic block consisting of three layers with each layer having a 450 voxels; 30 × 15 (*x* × *y*), is constructed. After structure modeling, additional forces and constraints are applied to generate a final deformation. Figure 3b shows the example block as fixed to the ground on both short ends (green rectangles), and a block of force is applied in the positive y direction (purple prism). Depending on the material properties of the soft structure and the mechanical stimulus range, VoxCad calculates the final posture of the object as shown in Figure 3c with a color coding where lighter colors represent higher magnitudes of positive strain. In our approach, we divide the selected mechanical stimulus range into seven equal steps and generate final postures of the objects for each step. These consecutive postures are collected together to form a complete set for the whole stimulus range.

### 3.2. Strain Vector Extraction

We developed a plug-in for VoxCad in order to extract the strain information of every voxel in a vector form, which we call “strain vectors”. For a soft structure model consisting of *n* voxels, the strain vector if the *i*^th^ voxel, *V_i_*_,_ has the following format:

(2) $$V_{i}=\left\lfloor s_{x},s_{y},s_{z},p_{x},p_{y},p_{z}\right\rfloor$$

where *s* is the magnitude of strain and *p* is the position of the voxel in three axes. Therefore, the final posture of a soft structure due to a selected deformation generates a strain matrix of size *n* × 6. Figure 3d shows the resulting strain vectors on the topmost layer of the block in the given example. In the detail, the position of a vector and its magnitude in the *x*-*y* direction can be seen as well. In our approach a complete deformation set is represented with consecutive seven steps which eventually generate a strain matrix of size 7 × *n* × 6 which is denoted as M.

### 3.3. Localization of Strain Regions

After the strain matrix is generated, the strain vectors in this matrix are analyzed to localize the characteristic strain regions of the deformation. In our method, we concentrate on the surface layer of the whole structure to comply with easy sensor attachment. Voxels on the surface layer is found by analyzing their “*p_z_*” value. This reduces the size of the multidimensional strain matrix into |M′| = 7 × *m* × 6, where *m* = *n*/*q* and *q* is the number of layers in the soft structure. In order to find characteristic regions on this topmost layer, direction information of the strain vectors are used. For every strain vector in the reduced matrix M′, the angle of strain direction in the *x*-*y* plane, *i.e.*, θ*_i_*, is found by:

(3) $$\theta_{i}=\arctan(p_{y}/p_{x})$$

whose physical explanation can be seen in the detail of Figure 3d. The resulting matrix with size 7 × *m* contains the angle information of every strain vector in all of the seven steps of the deformation. This matrix is then given as an input to MATLAB's K-means clustering tool [43] which uses a similar algorithm that was originally suggested [44]. The main idea behind this clustering method is to find a “*k*” number of discrete clusters, *i.e.*, S* = {*S*_1_, *S*_2_, … *S_k_*}, within a larger set, that are distinct from each other with respect to a defined set of properties:

(4) $$S*=\underset{S}{\arg\min}\sum_{i=1}^{k}\sum_{\theta_{j}\in S_{i}}{\|\theta_{j}-\mu_{i}\|}^{2}$$

Equation (4) shows the general approach of finding this discrete cluster set S*, where *k* is the number of clusters, θ*_j_* is the angle of strain, and μ*_i_* is the average of points, *i.e.*, average of strain angles, in *S_i_*. However, when only θ is used to generate the cluster set S*, it is possible that voxels belonging to a single cluster can be physically separated from each other by different voxels. That is why, in order to generate distinct regions in means of physical location and strain angle, we divide the cluster set S* into groups called “strain regions”, *i.e.*, *R_i_*, and generate a region set *R* = {*R*_1_, *R*_2_, … *R_l_*} where *l* ≥ *k*.

As explained in Algorithm 1, when the physical locations of voxel groups are taken into consideration in addition to θ, a larger region set *R* is formed. This set is composed of *R_i_*, which is a 7 × *m* matrix that represents the set of voxels that are physically next to each other and members of the same *S_i_*. Final representation of these strain regions can be seen in Figure 3e with different colored groups.

**Algorithm 1** Strain region generation algorithm.
**Data:** cluster set S*, reduced matrix M′**Result:** region set *R***for** every deformation step in M′ (1 to 7) **for** every voxel *V_i_* in M′ (*i* = 1 to *m*)   check all physical neighbors of *V_i_*;   **if** neighbor *V_j_* ∈ *S_i_*     make neighbor *V_j_* ∈ *R_i_*;   **else**     make neighbor *V_j_* ∈ *R_j_*;   **end** **end****end**

When strain regions are localized, we use their geometric properties to design the morphologies for strain sensors. Because of the fact that the strain sensors we use in this paper are fiber shaped, we start this task by conceptualizing the strain regions as template lines. As every strain region can be considered as a polygon which consists of several voxels whose strain vectors' angle are very similar to each other, each of these regions can also be represented by a single line which spans across the polygon with a slope of that region's average strain angle. In order to find the middle point of that line, we calculate the centroid point of a single strain region *R_i_* as:

(5) $$C_{i}=\frac{p_{x_{1},y_{1}}+p_{x_{2},y_{2}}+\cdots +p_{x_{r},y_{r}}}{r}$$

where *p_x_*_,_*_y_* represents the *x*, *y* positions of each voxel in the region *R_i_* with size *r*. For simplicity we omit the regions whose |*R_i_*| < 3 and centroid point is located out of its polygon. After that, we find the average strain magnitude in that region as follows:

(6) $$M_{i}=\sum_{j=1}^{r}\frac{\sqrt{s_{x_{j}}^{2}+s_{y_{j}}^{2}}}{r}$$

*M_i_* is used as a scaling factor to find the longest possible straight line that crosses the *C_i_* point. For simplicity we omit those regions whose *M_i_* < 0.05 mm as the strain will be weaker than detectable values. Eventually every region *R_i_* results in forming a line *l_i_*, which represents a suitable sensing location in that region, with length *L_i_*, and slope the same with region's average strain direction angle.

### 3.4. Sensor Pathway Planning

At this point of the approach, we have a set of regions *R_i_* and a set of lines *l_i_*, which represent a suitable sensor location for every region. We hypothesize that a final pathway which is a result of the connection of a combination of these lines will yield the morphology of a strain sensor that can distinctively represent that soft structure's deformation. Therefore, we use a path planning algorithm which connects these lines by using a cost function to determine the final pathway for the sensors. We define the cost function for any voxel with a position [*p_x_*, *p_y_*] on the topmost layer, to be connected to the representative region line *l_i_* of any *R_i_* as:

(7) $$f(i)=d/\left({L_{i}}^{*}M_{i}\right)$$

where *d* is the Euclidean distance between that voxel and the closest point on the region line *l_i_*. Equation (7) basically suggests that any line with high strain magnitude or length will yield a lower cost, and therefore will be preferred in the path planning algorithm.

Our path planning algorithm starts with the initialization of the end points of the final sensor pathway. When these points are defined, the algorithm basically searches every possible *l* to find a final pathway that connects the start point to the end point with minimum cost with respect to the cost function. As shown in Algorithm 2, the algorithm starts on the start point and moves towards the end point by connecting the lines together until either there are no more possible lines in front of it or the end point is reached. When either of these situations holds, the algorithm finalizes the set PW which basically consists of the geometric information about the chosen lines.

**Algorithm 2** Weighted Cost Path Planning Algorithm.
**Data:** region set *R* and lines *l***Result:** final pathway PWinitialize points [start, end] on the surface;set current point P to [start];**while *R*** ≠ Ø **or *P*** ≠ [**end**] **do**   check all *R_i_* in *R*;   find *R_i_* with min *f*(*i*);   add *R_i_* to PW;   update *P* with end point of *l* of *R_i_*;   remove *R_i_* from *R*;**end**

When the lines in the PW set selected by Algorithm 2 are connected to each other, the final pathway is determined. This pathway consists of two major parts; region lines *l*, as in PW, and straight connection lines which connect them together. While the strain information presented by region lines are true and shows the characteristics of selected regions, connection lines might produce erroneous strain information as they can go through several regions by ignoring strain direction angles. This classification enables us to emphasize true strain information during sensor output estimation. The resulting shape of the final pathway can be seen in Figure 3e, where region lines are shown with complete lines and connection lines are shown with dashed lines.

### 3.5. Sensor Modeling

The pathway can be considered as the final form for sensor morphology, as we are using thin, fiber shaped strain gauge sensors which could be laid directly on this pathway. Therefore, also considering CTPE's elastic properties and linear response to applied strain, we can use the strain information collected from this pathway as a mean to estimate sensor output *O* as:

(8) $$O={L_{PW}}^{*}K_{B}+N*W_{r}*K_{S}+W_{c}*K_{S}$$

where *L_PW_* is the total length of the pathway, *W_r_* and *W_c_* are the total strain magnitudes gathered from the region and connection lines by using Equation (6), *K_B_* and *K_S_* are the base resistance and sensitivity values for CTPE-based fiber shaped sensors as explained in Chapter 2. Due to the line classification explained earlier, we can enhance the true strain response by simply multiplying by *N*, which physically means to add *N* − 1 lines in parallel with that original region line. Although it is possible to detect positive and negative strain with CTPE-based strain gauge sensors when they are integrated into structures with a pre-stretch [38], in our paper we choose to integrate these sensors with their resting form which allows us to detect only positive strain. Therefore, in the sensor modeling part we only use positive strains to ensure correct sensor output.

## 4. Experiments

In this paper, we perform experiments in simulation and physical platforms to discover the efficacy of our suggested method. For this purpose, we have chosen a scenario where discrimination of three soft deformation patterns; *i.e.*, bending, twisting and pushing, is aimed. In order to perform discrimination, sensor morphologies are designed and evaluated by experiments in both simulation and physical platforms. In first part, simulation experiments are performed to show the scalability of the method on various shapes of soft structures, given the current state of path planning and sensor placement algorithms explained in Section 3. In the second part, experiments on physical platforms are performed to compare with simulation results for the investigation of simulation limitations such single-material physics, linear elasticity assumption and limited data point collection.

### 4.1. Simulation Results

**Figure 4.**
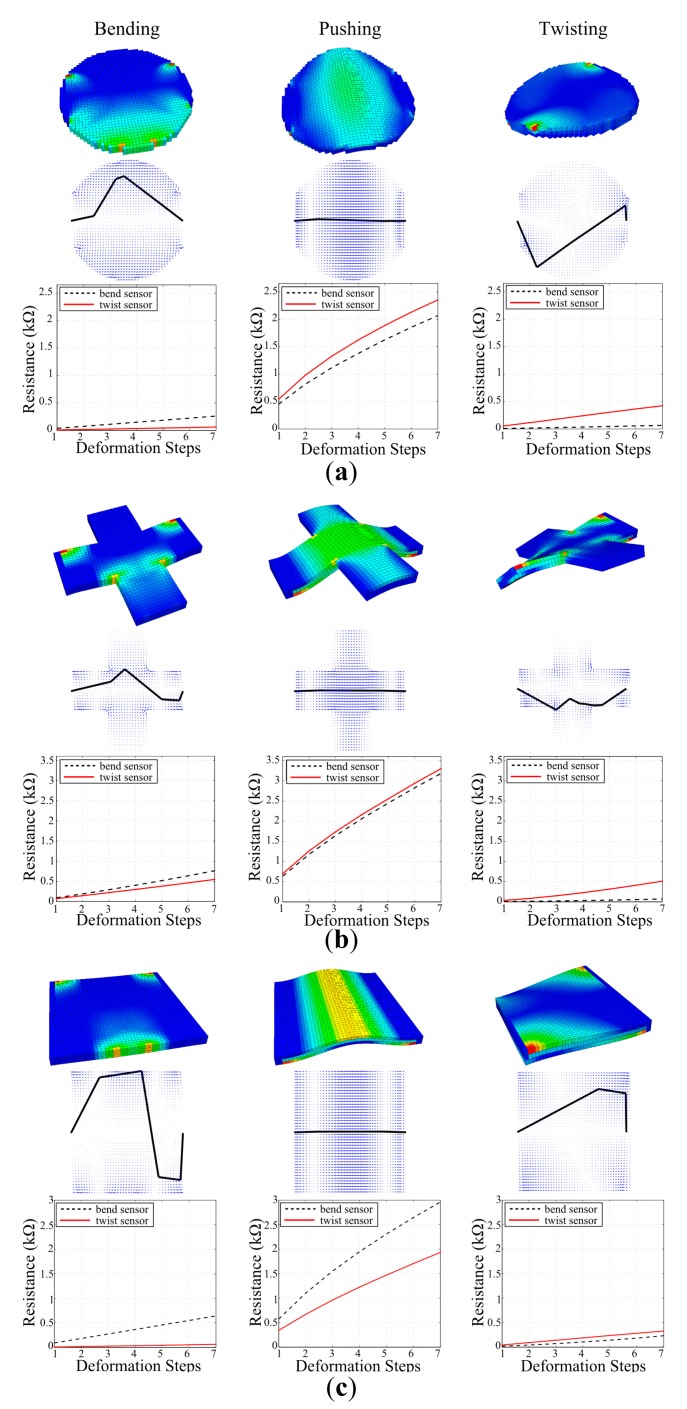
Simulation experiments of three shapes. Columns represent bending, pushing and twisting deformations respectively. First rows show VoxCad deformations with lighter colors representing larger strain and second rows show strain vectors with black lines representing designed sensor morphologies. Third rows show simulation estimates of sensors using Equation (8) with CTPE material's sensor properties. Deformation steps correspond to 1N of increase for bending and pushing, and 1 Nmm for twisting.

We start by modeling three different shapes aiming to show the scalability of the general approach given the current path planning algorithms. For this purpose we have chosen a circle, a plus and a square forms for simulated soft structure models. For fair comparison, similar sizes are chosen: all of the soft structures have three layers of voxel surfaces, with each voxel having a cubic shape of 1.5 mm in size, while the square and plus having 45 voxels on each side and circle having 45 voxels on its diameter. The structures are given linear elastic properties and constructed with the material properties of Silicone with a Young's modulus of 1.31 MPa. Each of these structures has undergone three different deformations, *i.e.*, bend, twist and push. For bending and pushing, a force range of 1–7 N and for twisting a torque range of 1–7 Nmm is applied. For every step within the range, the silicone blocks deformed with a gradual increase, and the postures they reached were captured and their strain vectors were extracted. The first rows of Figure 4a–c show the final postures of these blocks with the highest value in the applied range, where the first column is bending in the positive y direction, the second column is pushing in a positive z direction, and the last column is twisting around the positive x axis.

When the original vectors are used for region localization and sensory pathway planning, it is possible for the final sensor morphologies to have common parts, which can be a disadvantage for discrimination tasks. For a sensor to discriminate one specific deformation from the others, the number of these possible common parts should be kept at minimum. That is why we used these original strain vectors and performed a vector subtraction. The subtraction increases the chances of the elimination of common regions as the resulting vector properties for such regions will yield either very small magnitudes or negative directions which will be ignored during pathway planning. For our case, we subtracted bending and twisting from each other, and used the original vectors for pushing deformations. Following this method, we localized the strain regions and generated the final pathways which eventually created three unique sensor morphologies for each of the deformations. The resulting sensor morphologies can be seen in the second rows of Figure 4a–c.

The last rows of Figure 4a–c show the performances of sensor morphologies designed for bending and twisting, when they are tested on each deformation. It could be seen from the first columns of sensor estimation figures that the sensor designed for bending exhibits a larger response than the sensor designed for twisting in case of bending deformation. The reverse of this claim also holds for the twisting sensor for twisting deformations as it can be seen in the last columns of estimation figures. This is a valid indication that when strain vectors are subtracted from each other for discrimination tasks, the final sensor morphologies are different from each other, and they perform distinctively in their corresponding deformation tasks.

The designs of these sensors in the second rows of Figure 4a–c also validate the approach as bending and twisting sensors do not share common pathways. The sensor morphologies of sensors for only pushing task also confirm this as we only used the strain vectors of pushing deformations during their design, *i.e.*, the vector subtraction method for eliminating common regions with other deformations were not used. This generated a sensor morphology which is approximately a straight line connecting the start and end points together, as there is a single dominant strain region with positive strain in the middle section of the shapes. The middle columns of the last rows of Figure 4a–c show this dominant region as a dense collection of vectors with an average θ = 0°. When we look at the response estimates of bending and twisting sensors for pushing deformation for all structure shapes, we see that all generate a much larger response compared to their dedicated task. This is mainly due to the greater strain originated in the pushing deformation compared to the others. When the strain vectors of each deformation are investigated in Figure 4, it can be seen that pushing deformation generates a larger surface with a stronger strain. This can be understood by the density of blue colored vectors under the final pathways. Compared to bending and twisting, where distinctive strain regions are scattered around the surface because of vector subtraction and with lower strain magnitudes (can be seen by the lightness of the blue colored vectors), the pushing deformation creates a dominant strain region in the mid-section of the structure surface with high magnitudes. Therefore, when Equation (8) is applied to estimate sensor responses, both sensor designs generate a higher output in pushing deformation relative to others. Even though pushing deformation was not included in the design of sensor morphologies for discriminations, when we look at the estimation figures we can see that both of the sensor responses combined can be used to discriminate all three deformations.

Results shown in Figure 4 show that SVAS^3^ method can be applied to various shapes of soft structures to generate sensor morphologies for discrimination tasks. In this means, we can claim that our method is scalable to different structure shapes as long as the used sensor localization and path planning algorithms explained in Section 3 are given.

### 4.2. Experiments on Physical Platforms

While simulations only can show that SVAS^3^ method can generate sensor morphologies and estimate the sensor performances, the influence of limitations on single-material physics, linear elastics assumptions and limited number of data points need to be explored by physical experiments. For the validation and evaluation of these aspects, we performed two sets of experiments on physical platforms following simulations and tested the designed sensors. In the first set, the deformation scenarios in the simulations explained in Section 4.1 are tested to evaluate the applicability of the design method and the scalability on different structure shapes. In the second set, the effect of single material physics and other simulation parameters such as line multiplication is evaluated on a rectangular silicone block.

**Figure 5.**
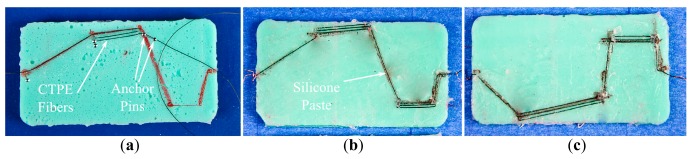
(**a**) The integration step of CTPE-based strain sensors on the molded silicone blocks. The designed and realized sensor morphologies for bending (**b**) and twisting (**c**).

In order to validate the performance of designed sensor morphologies in the earlier simulations, we molded several silicone (Mold Max^®^40 Series, E = 1.31 MPa) blocks with the same size in the 3D models. We built silicone blocks with the shape of circle, plus and square for the first experiment set, and rectangular blocks for the second set. Then we fixed fiber shaped CTPE-based strain gauge sensors on the silicone blocks by following the designed pathways. In order to place the sensor fibers accurately on the guidelines with no slack, we used steel pins as anchor points on the silicone and attached these sensors to the silicone block surfaces with a high elasticity transparent silicone glue (Dow Corning 732). Figure 5a shows the integration process for the second experiment set where CTPE-based sensors are stretched over the rectangular silicone block by anchor pins and then attached to the surface with the silicone paste.

To induce deformations for bending, twisting and pushing, we constructed three different experimental setups. For bending, a clamping mechanism is created to fix both ends of the silicone blocks to the ground. Another clamp is attached to the center of the silicone block and connected to a servo motor which produces linear force in positive y direction. A linear force gauge is placed in series within this connection to measure the applied force.

**Figure 6.**
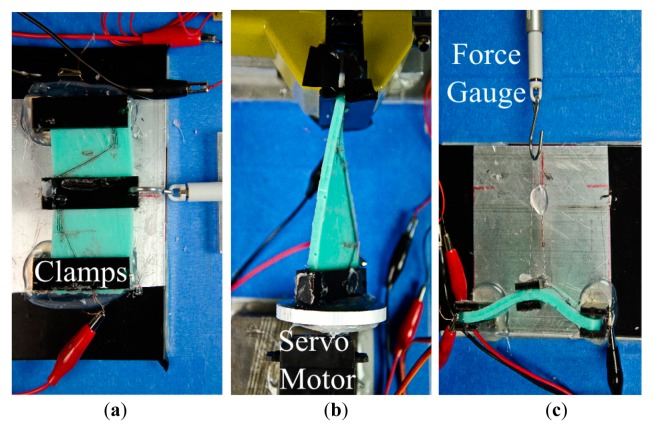
Experimental setups which generate deformations for (**a**) bending; (**b**) twisting and (**c**) pushing.

A similar setup is used for pushing, only for the exception that the force was applied in the positive *z* direction. For the twisting, a clamp is attached to fix one end of the silicone block. The other end is attached directly to a servo motor shaft to generate torque. Total amount of twist angle is measured with an angle compass and values are mapped into torque. The forces and torques are applied continuously with an increase of 1 N and 1 Nmm every 0.5 s. In all setups, CTPE sensors are connected to a simple voltage divider circuit, whose output is processed with an Arduino Due^®^ microprocessor. Figure 6 shows setups for both experiment sets.

**Figure 7.**
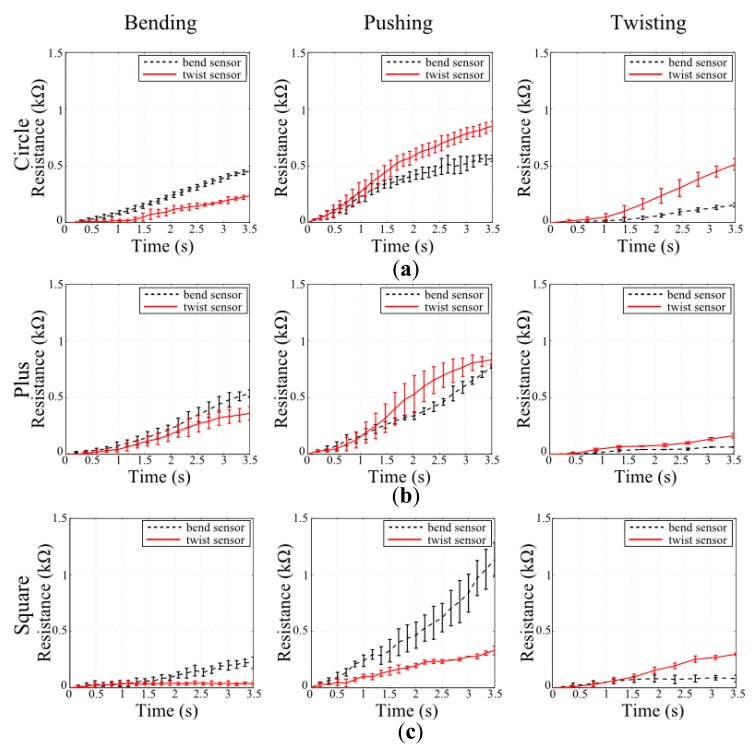
Experimental results of designed sensors for bending and twisting deformations on different structure shapes. In every shape, two sensor designs; *i.e.*, bend and twist sensors, are tested for three deformations. While each row shows the experiments on structure shapes, every column shows the deformation type. Experimental setup provides steps of stimulus increase for every 0.5 s during deformations; 1 N for bending and pushing, and 1 Nmm for twisting.

The first set of experiments investigates the applicability of the design method on physical platforms and its scalability on various structure shapes. Figure 7 shows the experimental results done with the sensor designs provided in simulations in Section 4.1. It can be seen from the figure that the quality of the sensor performances follows the simulation estimates in Figure 4. In other words, the sensors designed for their corresponding deformation; *i.e.*, bend sensor for bending deformation, generate a larger respond than the other sensor when that particular deformation is applied. The experiments not only suggest that the proposed design methodology for sensor morphologies can be applicable in real world, but also show that the designed sensors can be applied to various structure shapes as suggested by the simulations. In compliance with the simulation estimates, the first set of experiments also show that when a certain deformation is not considered as a contributing factor during the sensor design, the generated sensor morphologies' performances on these deformations cannot be predicted or programmed. This result can be seen in both simulation and experimental results when the performances of bend and twist sensors are investigated in pushing deformations.

While the first set of experiments support the general applicability of the sensor designs, an additional set of experiments were required to investigate the impact of single material physics and line multiplication parameters of the simulations on physical implementations. For this reason we generated a similar simulation scenario to test these factors. Here, we started with simulating a prismatic block similar to Figure 4c which is composed of three layers with 1800 voxels; 60 × 30 (*x* × *y*), in each layer. Every voxel is cubic shaped with a side length of 1.5 mm, making the size of the complete block as 90 mm × 45 mm × 4.5 mm. In order to investigate the material effect, the block is simulated with two different materials with linear elastic properties and Young's modulus of 1.31 MPa and 2 MPa. Additionally, the characteristic region lines, as explained in Equation (8), on the sensor morphologies are multiplied with 3 to enhance the sensor response. We have applied the same force ranges as in the previous section, generated the exact same deformations and designed two sensor morphologies.

**Figure 8.**
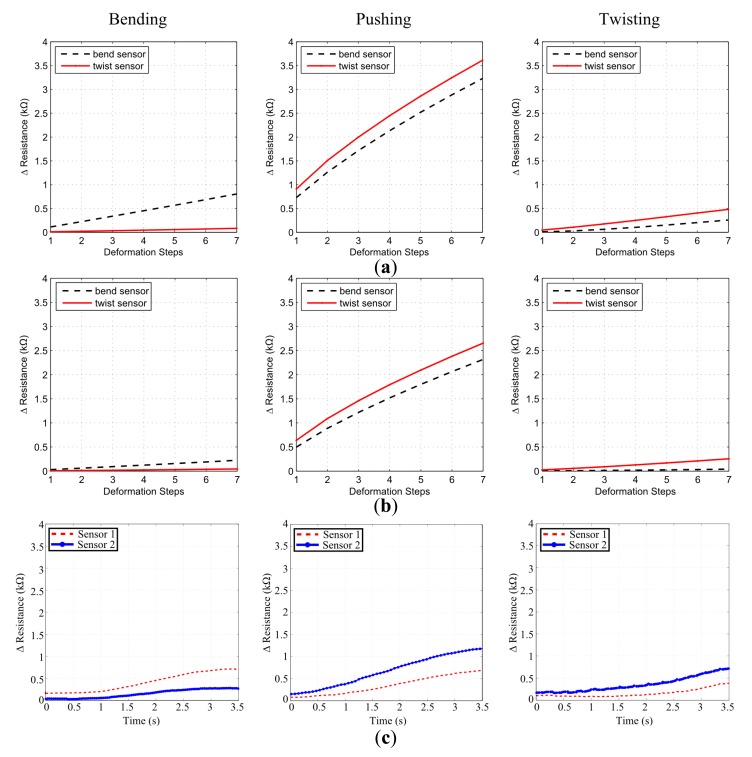
Case study on posture discrimination: twist, bend and push patterns. Simulation estimates for (**a**) block of silicone (E = 1.31 MPa); (**b**) block of CTPE (E = 2 MPa) and (**c**) experimental results with silicone (E = 1.31 MPa). Deformation steps in (a) and (b) correspond to every step of stimulus increase in simulations; 1 N for bending and pushing, 1 Nmm for twisting. Experimental setup provides same continuous increase for every 0.5 s in (c).

We can see that the experimental result in Figure 8c follows the trend suggested in the simulation estimates qualitatively in Figure 8a,b. Sensor 1, which was designed for bending deformation outperforms Sensor 2 in bending, while the reverse of this case hold for Sensor 2, which was designed for twisting, in twisting deformation. Additionally, both of the sensors generate a larger response in pushing deformation as suggested by the simulation. In order to ensure that twisting and bending can be detected by these sensor designs, we have run additional experiments and showed that sensor responses are significantly different. We repeated each of the deformations five times and collected the average peak values of Sensors 1 and 2 responses in bending and twisting experiments. For the bending tests, Sensor 1 had an average peak response of 0.688 ± 0.019 kΩ and Sensor 2 had 0.263 ± 0.008 kΩ, whose difference yields a *p* value of 7.61 × 10^−7^ in a standard *t*-test for statistical significance. Similarly, for the twisting tests Sensor 1 had an average peak response of 0.365 ± 0.027 kΩ and Sensor 2 had 0.682 ± 0.004 kΩ whose difference yields a *p*-value of 1.26 × 10^−5^. As both of these final *p*-values are lower than 0.01, the experiments show that the designed sensor morphologies for the specified task can achieve successful discrimination.

## 5. Discussion

### 5.1. SVAS^3^ Evaluation

In the current state of our approach, we model soft structures with a single type of material and use the strain information from the deformation defined by this material's properties. As we only use this strain information for sensor response estimation, sensor outputs are directly dictated by properties of the material. As it can be seen in Figure 8a the block simulated with CTPE material properties generated a lower strain; therefore lower sensor response, compared to a higher elasticity silicone material Figure 8b. However, in the physical platform, three different types of materials are involved throughout the sensing process which changes the output of sensors. We know that when fiber shaped CTPE sensors are placed onto the silicone structure and attached with another silicone paste, each of these structures will undergo a different amount of deformation. This can be explained by Equation (1) as the Young's modulus of each of these materials is different from each other, which influences the amount of strain they will exhibit under same amount of stress. Figures 7 and 8c show that in reality, the sensor output shows some discrepancy compared to simulation experiments due to this aforementioned multi-material interaction physics. Additionally, the manual integration process is also error-prone as slacks or disconnected parts in between the CTPE-based fibers and the silicone blocks can occur which can change the sensor output due to physical interaction.

While we look at the sensor outputs in experimental cases, we see that the curves have a non-linear tendency unlike the simulation estimates. There are two major contributing factors for this difference. The most influential factor is the amount of data points collected in the simulation estimates. For a complete stimulus range, there are seven data points for all of the deformations. An estimate depending on this amount of data points influence the final sensor output to have an almost linear trend. As experimental results reveal that sensors actually have non-linear output trends, it shows that collecting more data points within a stimulus range can capture the sensor behavior more correctly. Also the linear elasticity assumption for material models in our simulations influences the sensory output. Similar to the lack of multi-material physics in our simulation, the linear elasticity assumption is also an effective limiting factor for the current state of our approach.

The path planning algorithm as described in Algorithm 2, also influences significantly the final sensor morphology, therefore the sensor output. In our paper we chose a straightforward planner which uses Equation (7) as its cost function while it connects possible region lines to each other. Although this cost function guarantees the selection of region lines with higher magnitude or length, it does not specifically consider the length of the connection lines. Connection lines can span across multiple strain regions disregarding their strain direction just for the sake of connecting the ends of region lines. When a strain sensor is placed on top of these lines, it can pick up strain information from multiple regions which were actually eliminated in vector subtraction method. This creates irrelevant strain information which disturbs the quality of sensor performance.

### 5.2. Possible Future Application

So far we have shown the details of the SVAS^3^ approach and tested its designs on generic soft deformable blocks. Regarding the scalability of the general method and ease of applicability of the sensorization using CTPE-based sensors, we claim that this solution can be used in many fields such as wearable electronics, smart textile and especially robotics.

Here we show that our approach can be applied to a simple glove to discriminate hand signs from American Hand Sign Language [45] which represent letters “E”, “T” and “H”. In addition to the obvious reason, these letters are selected due to characteristic postures of metacarpophalangeal and proximal interphalangeal (first and second joints from the base of the finger) joints of the middle finger. We have simulated a hand model and gestures to generate these selected letter hand signs. Our simulations also have chosen these locations for the sensors to perform successful discrimination.

**Figure 9.**
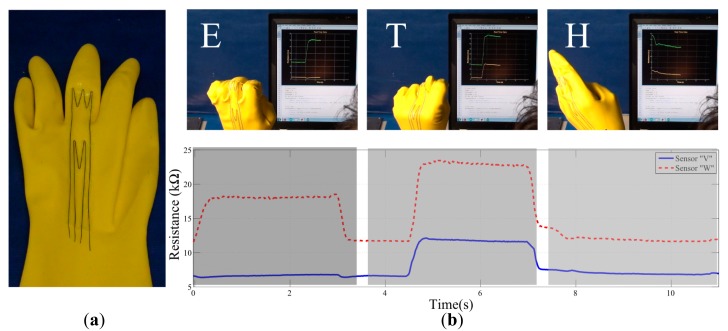
Experiments with CTPE sensors attached on plastic gloves to detect complex hand postures (**a**); In our case, American hand sign language is used to discriminate letters “E”, “T” and “H” in the experiments (**b**).

To evaluate the sensors, we have used a commercially available water sealant glove (Mapa-Pro^®^ Alto 258) made out of natural latex. We placed the CTPE-based sensors in the same way as in previous experiments using the silicone paste and pin anchors. In order to show the potential use of CTPE-based sensors and our flexible morphology design, we have taken the initiative to re-rout the sensory pathways to start and end at the same location on the wrist. This enabled cabling interface of the sensors to be centralized in the same region to allow more flexible and comfortable operation. However, as the current state of our approach does not suggest this re-routing, the final morphologies of the sensors on the chosen joint locations are designed by the authors. We decided to apply the signal enhancement by line multiplication option by placing the part of the sensors in a “W” and “V” shape on the characteristic strain regions on the finger joints. Figure 9a shows snapshots from the final morphology of sensors in experimental setups. The end points of the sensors are then connected to a simple voltage divider circuit, whose output is processed with an Arduino Due^®^ microprocessing unit.

The experimental results clearly present the potential use of this approach. Initially it can be seen that the designed sensors have a unique response to each of the letters, which can be easily used for discrimination. For clarity, we will call the sensor that span through first and second joints as “Sensor W” and the other shorter sensor as “Sensor V” with respect to the shapes they have on the joints.

When the response to each letter is investigated, several different implications can be perceived. For the letter “E”, we see that only sensor W responds as only the second joint of the middle finger flexes. In letter “T”, both of the sensors respond due to the flexion of both joints, however the magnitude of Sensor W's response is nearly the double of Sensor V, as it spans through both joints. This is a good indicator that, by using CTPE-based continuous sensors, complex responses can be achieved even with a single sensor and multiple postures can be discriminated as the sensor output combination will no longer be discrete. When we look at letter “H”, we see that none of the sensors respond as there is no strain on any of the joints.

We also see that the responses of the sensors are very fast with respect to the motion as well. This is generally due to the relationship between the sensor's and target structure's elasticities. As long as the elasticity of the target platform is lower than the sensor, the total amount of strain will be dictated by the structure and the deformation of the sensor will be controlled by it. This will result in a more robust and reliable sensory data to be gathered.

## 6. Conclusions/Outlook

In this paper we have proposed a novel approach named SVAS^3^ which designs flexible sensor morphologies by using the strain information generated in soft deformations. In this context, our method involves simulation tools to model soft structures and deformations to extract necessary strain information to construct sensor morphology designs. We have chosen a carbon black/thermoplastic elastomer material (CTPE) to model and generate strain gauge sensors with linear sensitivity response characteristics. The current state of our method models fibrous strain gauge sensors and uses extracted strain information to design custom pathways for these fibers to follow. In order to show the scalability of our approach to various soft structure materials and applications, we have performed simulations and experiments to discriminate complex behaviors. We have generated two sensor morphologies by using our method to discriminate three postures on various shapes of silicone blocks due to bending, twisting and pushing deformations. To validate the efficacy of our approach and sensor performance estimations, we have casted different shaped blocks out of silicone, fabricated fiber shaped CTPE sensors and integrated them following the morphology designs generated by the simulations. By comparing the simulation and experimental results, we confirm that the proposed approach is able to discriminate the three motion patterns with tunable performance. We also proposed the application of this method in other research fields by showing an example case on gloves to discriminate American Hand Sign Language based “E”, “T” and “H” letters. With respect to the current state of our approach, we used sensor locations suggested by our simulation method and experimentally applied the sensor morphologies based on the simulation results. The experiments showed successful discrimination results as well as the potential of the use of our approach for more complex applications. Overall, we showed that our approach can design sensor morphologies by simulating soft deformations and estimate sensor performances which are validated by following experiments. Such a sensor design approach can have an impact on sensor morphology for detecting complex behaviors and postures for soft continuum bodied structures. The usage of CTPE as a material for the fabrication of strain gauge sensors also supports this idea, as many different morphologies can be created and easily integrated into soft structures.

The comparison of simulation and experimental results still shows a quantitative gap between simulation and experiments that should be closed using a multi-material physics approach in the future and by the investigation of non-linear elastic models, sensors hysteresis and drifts. Another aspect could be a more detailed analysis on the simulation parameters such as threshold values and limits used in decision making, region clustering and path planning algorithms. The effect of voxel resolution, the size of the target structure and the shape limits could be discussed even further to investigate the limits and scalability of our approach. Similarly, different path planning algorithms could be investigated to maximize sensor response and improve discrimination performances by minimizing the amount of error generated by connection lines. Also collecting more data points in the simulations can capture the expected performance of the sensors.

In our paper we have chosen CTPE material due to our familiarity with the fabrication of strain gauge sensors with it in addition to its compatibility to our example applications in means of electrical and mechanical properties. However alternative state-of-the-art materials can also be investigated to model and fabricate strain gauge sensors for different applications. This will have a positive effect on the range of applications for these sensors as softer types of target platforms would generate more reliable results. Also alternative sensor embedding techniques such as printing and casting can be investigated as our method generates designs of flexible sensor morphologies which can be adapted by other methods as a design guideline. Finally, an extension to dynamics applications can be researched [46] to show the applicability to robotics field. Soft robots which require elastic and adaptive sensor systems can benefit from this approach to include embedded sensorization.

## References

[1] Trimmer B. (2013). Soft Robots. Curr. Biol..

[2] Majidi C. (2013). Soft Robotics: A Perspective—Current Trends and Prospects for the Future. Soft Robot..

[3] Brown E., Rodenberg N., Amend J., Annan Mozeika A., Steltz E., Zakin M.R., Lipson H., Jaeger H.M. (2010). Universal Robotic Gripper Based on the Jamming of Granular Material. Proc. Natl. Acad. Sci. USA.

[4] Lin H.T., Leisk G.G., Trimmer B. (2011). GoQBot: A Caterpillar Inspired Soft Bodied Rolling Robot. Bioinspir. Biomim..

[5] Laschi C., Cianchetti M., Mazzolai B., Margheri L., Follador M., Dario P. (2012). Soft Robot Arm Inspired by the Octopus. Adv. Robot..

[6] Shepherd R.F., Ilievski F., Choi W., Morin S.A., Stokes A.A., Mazzeo A.D., Chen X., Wang M., Whitesides G.M. (2011). Multigait Soft Robot. Proc. Natl. Acad. Sci. USA.

[7] Dobrzynski M., Camara R.P., Floreano D. Contactless deflection sensor for soft robots.

[8] Lee S., Suh I.H., Kim M.S. (2008). Recent Progress in Robotics: Viable Robotic Service to Human.

[9] Webster R.J., Kim J.S., Cowan N.J., Chirikjian G.S., Okamura A.M. (2006). Nonholonomic Modeling of Needle Steering. Int. J. Robot. Res..

[10] Webster R.J., Memisevic J., Okamura A.M. Design considerations for robotic needle steering.

[11] Kallem V., Cowan N.J. Image guidance control of flexible bevel-tip needles.

[12] Cianchetti M., Renda F., Licofonte A., Laschi C. Sensorization of continuum soft robots for reconstructing their spatial configuration.

[13] Vogt D., Menguc Y., Park Y.-L., Wehner M., Kramer R.K., Majidi C., Jentoft L.P., Tenzer Y., Howe R.D., Wood R.J. Progress in soft, flexible, and stretchable sensing systems.

[14] Lucarotti C., Oddo C.M., Vitiello N., Carrozza M.C. (2013). Synthetic and Bio-Artificial Tactile Sensing: A Review. Sensors.

[15] Dahiya R.S., Metta G., Valle M., Sandini G. (2010). Tactile Sensing—From Humans to Humanoids. IEEE Trans. Robot..

[16] Nambiar S., Yeow J.T.W. (2011). Conductive Polymer-based Sensors for Biomedical Applications. Biosens. Bioelectron..

[17] Chossat J., Park Y.-L., Wood R.J., Duchaine V. (2013). A Soft Strain Sensor Based on Ionic and Metal Liquids. IEEE Sens. J..

[18] Gibbs P.T., Asada H.H. (2005). Wearable Conductive Fiber Sensors for Multi-axis Human Joint Angle Measurements. J. NeuroEng. Rehabil..

[19] Muhammad H.B., Recchiuto C., Oddo C.M., Beccai L., Anthony C.J., Adams M.J., Carrozza M.C., Ward M.C.L. (2011). A Capacitive Tactile Sensor Array for Surface Texture Discrimination. Microelectron. Eng..

[20] Cheng M.Y., Huang X.H., Ma C.W., Yang Y.J. (2009). A Flexible Capacitive Tactile Sensing Array with Floating Electrodes. J. Micromech. Microeng..

[21] Hwang E.-S., Seo J.-H., Kim Y.-J. (2007). A Polymer-Based Flexible Tactile Sensor for Both Normal and Shear Load Detections and its Application for Robotics. J. Microelectromech. Syst..

[22] Beccai L., Roccella S., Arena A., Valvo F., Valdastri P., Menciassi A., Carrozza M.C., Dario P. (2005). Design and Fabrication of a Hybrid Silicon Three-axial Force Sensor for Biomechanical Applications. Sens. Actuators A Phys..

[23] Park Y.L., Chen B., Wood R.J. (2012). Design and Fabrication of Soft Artificial Skin Using Embedded Microchannels and Liquid Conductors. IEEE Sens. J..

[24] Ingber D.E. (2004). Mechanochemical Basis of Cell and Tissue Regulation. Biotechnol. Revolut..

[25] Dangles O., Megal C., Pierre D., Oliver A., Casas J. (2005). Variation in Morphology and Performance of Predator-sensing Systems in Wild Cricket Populations. J. Exp. Biol..

[26] Lichtensteiger L., Pfeifer R. An optimal sensor morphology improves adaptability of neural network controllers.

[27] Nurzaman S.G., Utku C., Brodbeck L., Wang L., Iida F. (2013). Active Sensing System with *in situ* Adjustable Sensor Morphology. PLoS One.

[28] Parker G.B., Nathan P.J. Concurrently evolving sensor morphology and control for a hexapod robot.

[29] Takei K., Takahashi T., Ho J.C., Ko H., Gillies A.G., Leu P.W., Fearing R.S., Javey A. (2010). Nanowire Active-matrix Circuitry for Low-voltage Macroscale Artificial Skin. Nat. Mater..

[30] Correia V., Caparros C., Casellas C., Francesch L., Rocha J.G., Lanceros-Mendez S. (2013). Development of Inkjet Printed Strain Sensors. Smart Mater. Struct..

[31] Noguchi Y., Sekitani T., Someya T. (2006). Organic-transistor-based Flexible Pressure Sensors Using Ink-jet-Printed Electrodes and Gate Dielectric Layers. Appl. Phys. Lett..

[32] Huang C.-T., Shen C.-L., Tang C.-F., Chang S.-H. (2008). A Wearable Yarn-based Piezo-Resistive Sensor. Sens. Actuators A Phys..

[33] Zhao H., Zhang Y., Bradford P.D., Zhou Q., Jia Q., Yuan F.-G., Zhu Y. (2010). Carbon Nanotube Yarn Strain Sensors. Nanotechnology.

[34] Yamada T., Hayamizu Y., Yamamoto Y., Yomogida Y., Izadi-Najafabadi A., Futaba D.N., Hata K. (2011). A Stretchable Carbon Nanotube Strain Sensor for Human-motion Detection. Nat. Nanotechnol..

[35] Cochrane C., Koncar V., Lewandowski M., Dufour C. (2007). Design and Development of a Flexible Strain Sensor for Textile Structures based on a Conductive Polymer Composite. Sensors.

[36] Costa P., Silva J., Sencadas V., Simoes R., Viana J.C., Lanceros-Méndez S. (2013). Mechanical Electrical Electro-mechanical Properties of Thermoplastic Elastomer Styrene–Butadiene–Styrene/Multiwall Carbon Nanotubes Composites. J. Mater. Sci..

[37] Melnykowycz M., Koll B., Scharf D., Clemens F. (2014). Comparison of Piezoresistive Monofilament Polymer Sensors. Sensors.

[38] Mattmann C., Clemens F., Troster G. (2008). Sensor for Measuring Strain in Textile. Sensors.

[39] Flandin L., Hiltner A., Baer E. (2001). Interrelationships between Electrical and Mechanical Properties of a Carbon Black-filled Ethylene–octene Elastomer. Polymer.

[40] Clemens F., Koll B., Graule T., Watras T., Binkowski M., Mattmann C., Silveira I. (2013). Development of Piezoresistive Fiber Sensors, Based on Carbon Black Filled Thermoplastic Elastomer Compounds for Textile Application. Adv. Sci. Technol..

[41] Fung Y.-C. (1977). A First Course in Continuum Mechanics.

[42] Hiller J., Lipson H. (2014). Dynamic Simulation of Soft Multimaterial 3D-Printed Objects. Soft Robot..

[43] (2012). MATLAB and Statistics Toolbox Release 2012b.

[44] Hartigan J.A., Wong M.A. (1979). Algorithm AS 136: A K-Means Clustering Algorithm. Appl. Stat..

[45] Liddell S.K., Johnson R.E. (1989). American sign language: The phonological base. Sign Lang. Stud..

[46] Culha U., Wani U., Nurzaman S.G., Clemens F., Iida F. (2014). Motion pattern discrimination for soft robots with morphologically flexible sensors.

